# Is There an “Ideal” Sequencing for Open Reduction and Internal Fixation of Multiple Mandibular Fractures with Condylar Neck Involvement? A Retrospective Cohort Study

**DOI:** 10.3390/jcm14207142

**Published:** 2025-10-10

**Authors:** Gian Battista Bottini, Wanda Lauth, Wolfgang Hitzl, Benjamin Walch, Maximilian Modelhart, Katharina Zeman-Kuhnert, Florian Huber, Florian Menapace, Marie-Christine Wilhelmstätter, Christoph Steiner

**Affiliations:** 1 Department of Oral and Maxillofacial Surgery and Center for Reconstructive Surgery, Paracelsus Medical University, 5020 Salzburg, Austria; g.bottini@salk.at (G.B.B.); b.walch@salk.at (B.W.); maxmodelhart@outlook.com (M.M.); k.zeman-kuhnert@salk.at (K.Z.-K.); fl.huber@salk.at (F.H.); f.menapace@salk.at (F.M.); mariewi123@gmail.com (M.-C.W.); 2 Team Biostatistics and Big Medical Data, IDA Lab Salzburg, Paracelsus Medical University, 5020 Salzburg, Austria; wanda.lauth@pmu.ac.at; 3 Department of Ophthalmology and Optometry, Paracelsus Medical University, 5020 Salzburg, Austria; w.hitzl@salk.at; 4 Research Program Experimental Ophthalmology & Glaucoma Research, Paracelsus Medical University, 5020 Salzburg, Austria

**Keywords:** maxillofacial trauma, condylar fractures, mandibular condyle neck fractures, mandibular fractures, facial trauma

## Abstract

**Background:** There is no consensus on the “best” sequencing for open reduction and internal fixation (ORIF) in multiple mandibular fractures involving the condyle. **Objective:** The objective of this study is to compare the outcomes between a “top-to-bottom” and a “bottom-to-top” ORIF sequence for multifocal mandibular fractures at the author’s institution. **Patients and Methods:** A retrospective cohort study of adult dentate patients with multifocal mandibular fractures treated with ORIF. Inclusion criteria were the presence of at least one condylar neck or basis fracture and one “non-condylar” mandibular fracture. The authors evaluated the reduction quality using radiological and clinical parameters, including the ramus/condylar neck angle, the presence of a gap at the lingual aspect in the dentate area, dental occlusion, the need for a redo operation, and the need for postoperative occlusal fine-tuning. **Results:** A total of 31 patients had a bottom-to-top sequence, 4 patients had a substandard outcome, 4 had an acceptable outcome, and 23 achieved an ideal outcome. Ten patients underwent a top-to-bottom sequence; one patient had an acceptable outcome, and nine patients achieved ideal outcomes. There was no significant difference between sequencing and outcome. (*p* = 0.231). However, the odds ratio for a suboptimal outcome regarding the bottom-to-top surgery as opposed to the top-to-bottom surgery was 4.80 (CI: 0.53–236.07). In other words, the odds of having a suboptimal outcome and a bottom-to-top sequence were 4.80 times higher than having a suboptimal outcome and a top-to-bottom sequence. **Conclusions:** Based on our results, the top-to-bottom ORIF sequence appeared to be a favorable factor.

## 1. Introduction

The mandible is exposed to trauma due to sports accidents, interpersonal violence, falls, and road traffic accidents. Multiple mandibular fractures involving the condylar process are a common finding, can be challenging to deal with, and represent a considerable burden for the patient and the healthcare systems [1,2,3,4,5]. However, despite the commonality of multifocal mandibular fractures, only a few studies have specifically addressed the question of whether there is an “ideal” open reduction and internal fixation (ORIF) sequencing for this fracture pattern [6,7,8,9,10,11]. The literature is controversial and consists of a small number of monocentric retrospective series, so one cannot rely on any convincing evidence. The present retrospective study aimed to compare outcomes between a “top-to-bottom” and a “bottom-to-top” sequence in two cohorts of patients who underwent ORIF for multiple mandibular fractures with condylar neck/base involvement. The authors aimed to identify an advantageous sequence for streamlining operative treatment and improving outcomes.

## 2. Patients and Methods

### 2.1. Study Design

The authors performed a retrospective cohort study including all adult patients with mandibular fractures treated by ORIF over eleven years from 1 January 2012 to 31 December 2022 at the Department of Oral and Maxillofacial Surgery, University Hospital of the Private Medical University Paracelsus, Salzburg, Austria. Formal ethics committee approval was not required, given the retrospective, non-interventional nature of the study. The data collected was pseudonymized. The study was conducted in accordance with the principles of the Declaration of Helsinki [12].

Inclusion criteria were the following: skeletally mature dentate patients with multifocal non-comminuted mandibular fractures involving the condylar neck/base on one or both sides with at least one additional “non-condylar” fracture at a more caudal level: angle, body, or symphysis/parasymphysis, and a minimum follow-up of 6 months.

Exclusion criteria included: conservative treatments, panfacial fractures, comminuted fractures, defect fractures, pediatric patients, multifocal mandibular fractures without condylar base/neck involvement, condylar head fractures, double condylar base/neck fractures without a third fracture at a more caudal level, single mandibular fractures, patients without adequate records and follow-up.

Condylar head fractures were excluded. Generally, the authors treat operatively non-comminuted condylar head fractures with loss of ramus height in dentate patients. However, many colleagues do not treat them operatively, regardless of the type of fracture. Secondly, the authors thought of creating more homogeneous groups, excluding condylar head fractures and limiting the analysis to the base and neck. Thirdly, the ramus/neck angle would not have served as a criterion to assess the quality of reduction, and the authors would have necessitated an alternative parameter instead. A fourth reason was that condylar head fractures can be significantly more challenging than base fractures, and it seemed reasonable to distinguish between them. However, during the retrospective analysis, the authors encountered and excluded a few cases of multifocal mandibular fractures with associated condylar head fractures that achieved excellent reduction by ORIF. The authors would recommend approaching them first, as for the condylar neck and base fractures.

Patients’ records provided demographic and clinical data. The authors reviewed patients’ documentation, including demographic data, diagnoses, fracture etiology, pre- and postoperative imaging, operative management, operative reports (with particular attention to the fixation sequence), follow-up, and complications as recorded in the clinical notes.

The primary independent (predictor) variable was the ORIF sequence.

Therefore, the patients who satisfied the inclusion criteria were divided into two cohorts according to the order of ORIF: from the symphysis/parasymphysis or angle to the condyle (bottom-to-top) versus from the condyle to the symphysis (top-to-bottom).

Secondary independent variables of interest were the surgeon’s expertise and the procedure duration.

The surgeon carrying out the procedure was either a trainee (junior) or a staff member with formal qualifications as a specialist (senior). However, for all patients in the present analysis, even when a trainee was performing the procedure, a senior was always present as an assistant.

The procedure duration was an indicator of the case difficulty for the surgical team.

The authors referred to the Loukota fracture classification and its subsequent modifications, adopted by the Arbeitsgemeinschaft für Osteosynthesefragen (AO) and the International Bone Research Association (IBRA). The classification is described extensively in the position paper from the IBRA Symposium on Surgery of the Head—The 2nd International Symposium for Condylar Fracture Osteosynthesis, Marseille, France 2012 [13,14].

Eckelt et al., in their seminal paper, considered condylar fractures with a deviation of 10–45°, or a shortening of the ascending ramus ≥2 mm, irrespective of the fracture level (condylar base, neck, or diacapitular/condylar) to be moderately displaced [15].

Therefore, the authors used a 45° angulation as a cut-off between acceptable and poor results, considering an angulation of less than 10° to be good.

In addition to the above classification and cut-offs, the authors established their quality indicators based on clinical experience.

The primary outcome was the quality of the surgical reposition, measured by the parameters of lingual gap, ramus/condylar neck angulation, and occlusion after open reduction and internal fixation. The primary hypothesis was that the top-to-bottom sequence would be superior to the bottom-to-top sequence in treating multiple mandibular fractures with condylar involvement.

### 2.2. Primary Outcome Measures (Primary Dependent Variables)

The angle between the condylar neck and the ramus after ORIF on the coronal view of a computed tomogram (CT) was measured. A postoperative angulation of less than 10° was considered good, 10–45° was considered acceptable, and >45° was considered insufficient.

**Figure 1:**
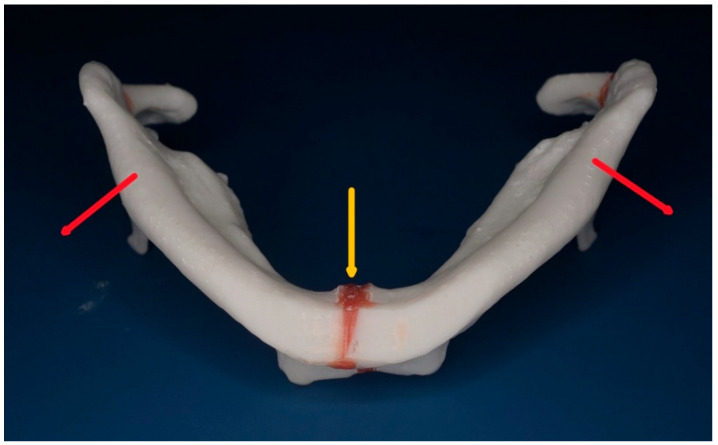
“Widening of the mandible”. Yellow arrow showing the splaying of the lingual cortical bone in a symphyseal mandibular fracture with concomitant condylar fractures (guardsman fracture). Red arrows: directions of the transverse widening of the mandible.

The presence of a gap at the lingual aspect (“lingual splaying”) in the symphysis/parasymphysis area or body after ORIF on the axial view of a CT scan. Less than 1 mm on the axial view was considered acceptable, and more than 1 mm was considered insufficient. When symphyseal/parasymphyseal or body fractures are plated with the buccal borders in contact, but the lingual borders separated (“lingual splaying”), the mandible “opens” similarly to a book with an increase in the intergonial distance and a broadening of the face. Some authors describe this phenomenon as a “widening of the mandible,” and it happens in the presence of a concomitant monolateral or bilateral condylar fracture (Figure 1). The broadening of the mandible may contribute to the so-called “dish face” appearance in the case of insufficient reposition after panfacial fractures.

The authors assessed the postoperative dental occlusion based on clinical findings and imaging, including cone-beam computer tomography (CBCT), panoramic (OPT), or computer tomography (CT). Clinically and radiographically, a return to the pre-injury occlusion or to a closed occlusion class I was considered ideal; a “slight” (<1 mm) open bite, if not present before the fracture, was deemed acceptable; an open bite of more than 1 mm was considered insufficient. The presence of a cross bite, if not present before the fracture, was also considered a marker of suboptimal reposition.

If at least two of the above parameters were insufficient, the overall surgical outcome was deemed substandard. If only one parameter was suboptimal, the overall outcome was considered still acceptable. If all the parameters were adequate, the overall outcome was judged as ideal.

A fourth quality indicator was the need for revision surgery, which in itself indicates a substandard surgical outcome.

A fifth and final element was the need to fine-tune the occlusion in the postoperative period. This aim was achieved using a variety of means, tailored to the individual occlusal findings. IMF was prescribed if the bite was open. Guiding elastics were used in cases of muscle incoordination to assist the patient in finding the maximum intercuspation. Selective occlusal grinding helped eliminate premature contacts, and orthodontic treatment was used to close the bite. All the above techniques indicated a suboptimal occlusion. However, they also implied an acceptable outcome from the surgical perspective, as the occlusal disturbances were minor.

### 2.3. Secondary Outcome Measures (Secondary Dependent Variables: Non-Occlusal Complications

Non-occlusal complications of interest included infections, neurologic sequelae such as numbness and palsy, temporomandibular joint dysfunction characterized by pain, limitation of mouth opening, limitations of lateral excursions, inability to return to a standard food consistency, and crepitus.

### 2.4. Measurement Methods and Software

Two authors independently performed measurements of multiplane reconstructions from cross-sectional imaging. For the lingual splaying, the authors rated gaps less than or equal to 1 mm as good. Gaps greater than 1 mm were considered as suboptimal. Regarding the neck-ramus angle, the authors similarly categorized neck-ramus angles as follows: less than or equal to 10 degrees as good, 11 to 45 degrees as acceptable, and greater than 45 degrees as suboptimal. Equally, the occlusion was assessed based on discrete categories such as closed bite (good), “slightly” open bite if less than 1 mm (acceptable), or open bite if equal or greater than 1 mm (suboptimal), cross-bite (suboptimal), need for postoperative occlusal “fine tuning” (acceptable), redo operation (substandard). In borderline cases, the authors reached a consensus among three of them.

For measuring, the authors used the ruler and protractor available in the tools section of the programs Deep Unity Diagnostics 2.0.2.2 (Dedalus Healthcare group, Milano, Italy) and Romexis 6.0 (Planmeca, Helsinki, Finland), which are the picture archiving and communication system programs (PACS) in use at their institution.

### 2.5. Statistical Analysis

For the descriptive analysis, mean and standard deviation were used for the metric variables, and the absolute and relative frequencies were used for the categorical variables. Fisher’s Exact test was used to examine differences between the bottom-to-top and top-to-bottom cohorts for nominal variables. For metric and ordinal variables, the non-parametric Mann–Whitney U test was applied. For a further and more comprehensive analysis, the outcome variables were binarized into two categories only, ideal versus suboptimal, merging the “acceptable” and “substandard” results into the category “suboptimal”. Furthermore, Fisher’s Exact test was applied for these categorized nominal variables, and, additionally, odds ratios with 95% confidence intervals were computed to compare the two cohorts. Each *p*-value is reported together with its corresponding effect size and 95% confidence interval. For 2 × 2 contingency tables, Fisher’s exact test was applied, and the odds ratio (OR) was calculated. For larger contingency tables, a global Fisher’s test was performed, and the strength of association was quantified using Cramér’s V with a bootstrap 95% confidence interval. For metric or ordinal variables, the Mann–Whitney U test was used, and the effect size was expressed as the standardized effect statistic r (derived from the Mann–Whitney U Z statistic), also with a bootstrap confidence interval.

A *p*-value smaller than 0.05 was assumed to be significant for all tests (two-sided). All calculations were carried out using the statistical software R (Version 4.3.2) [16].

The authors also performed a multivariable logistic regression model that included the number of fractures (two vs. three), surgeon expertise (junior vs. senior), and sequencing method (top-down vs. bottom-to-top). The authors did not include bilateral involvement, as nearly all fractures in the dataset were bilateral; therefore, this variable carried no discriminatory information. Similarly, time to surgery was excluded because all the patients were treated within 72 h. Fracture pattern/severity was also not included, since patient selection into the study was based on this characteristic. Excluding these variables also helped reduce the risk of overfitting, particularly given the limited sample size of the top-to-bottom group.

## 3. Results

Nine hundred and twenty-six patients were treated operatively for mandibular fractures in the 11 years between 1 January 2012 to 31 December 2022 at the authors’ department.

Forty-one patients showed the fracture pattern of interest and met the inclusion criteria. Of those, 9 (21.95%) were females, and 32 (78.05%) were males. Thirty-one patients were in the bottom-to-top cohort (75.61% of the whole sample), and ten patients were in the top-to-bottom cohort (24.39% of the whole sample).

### 3.1. Bottom-to-Top Cohort: Demographics and Etiology

Thirty-one patients were in the bottom-to-top sequence, comprising twenty-four (77.42%) males and seven (22.58%) females, with a mean age of 40.81 (SD 16.53).

In this cohort, the causes of the fractures were assaults (5 patients), falls (8 patients), and road traffic accidents (18 patients).

### 3.2. Top-to-Bottom Cohort: Demographics and Etiology

In the second cohort, 10 patients had their fractures repositioned and plated first at the condylar process, followed by the symphysis/parasymphysis or body (in a top-to-bottom sequence).

Eight (80%) were males and two (20%) females, and the mean age was 37.30 (SD 18.46).

In this cohort, the causes of the fractures were assaults (3 patients), falls (1 patient), road traffic accidents (5 patients), and work accidents (1 patient).

Patient’s demographics are summarized in Table 1 below. The difference between the sequencing methods in the age of the patients, as well as the association with sex, was not significant (*p* = 0.543, r = 0.10, CI: 0.01–0.43, and *p* = 1.00, OR = 1.16, CI: 0.17–13.72, respectively). Neither was the association with etiology, nor the difference in the number of fractures (*p* = 0.205, Cramer’s V = 0.34, CI: 0.09–0.63 and *p* = 0.514, r = 0.11, CI: 0.00–0.32).

### 3.3. Management Characteristics

All patients had an extended antibiotic prophylaxis for 7 days. Open reduction and internal fixation were carried out under general anesthesia with nasal intubation. The surgeons approached the condylar neck/base via a wide variety of approaches: “classic” submandibular, retromandibular, high submandibular with anteroparotid transmasseteric dissection (APTM), transoral endoscopic assisted, paramandibular, or preauricular incision, depending on the height of the fracture, the preference of the surgeon, and the choice of hardware. The above approaches are well described, and the interested reader is referred to three excellent books that analyze several approaches to the condyle and the mandible in detail [17,18,19]. To preserve the facial nerve during extraoral approaches, surgeons used loop magnification throughout the procedure. Nerve stimulation with a disposable nerve locator was employed to confirm or exclude the exact location of a facial nerve branch (Neuropacer, FIAB, Florence, Italy). As the authors’ department is located in a teaching hospital, a large, heterogeneous group of surgeons with varying levels of expertise is employed. Apart from a few young trainees, staff doctors operate most cases with loops and headlights. Therefore, it is a common practice to use loops when operating mandibular fractures. Loops can slow things down, but hopefully reduce the chance of injury to a fine branch of the facial nerve. To reduce the risk of facial palsy, a nerve stimulator may also be helpful in extraoral approaches. A final consideration could be made that, in the event of medico-legal issues, employing adjuncts as those above could be used as an argument to demonstrate the goodwill of minimizing the occurrence of facial nerve injuries. Nevertheless, it is absolutely possible to perform these procedures without magnification or nerve stimulation if one feels comfortable and confident doing so.

The anesthetist was instructed to withhold muscle paralytic agents during the dissection until reaching the bone, so that patients’ grimacing would warn the surgeons in case of inadvertent stimulation of the facial nerve branches. At the authors’ department, there has been a transition over the years from the “classic” submandibular approach to the “high” submandibular approach.

**Figure 2:**
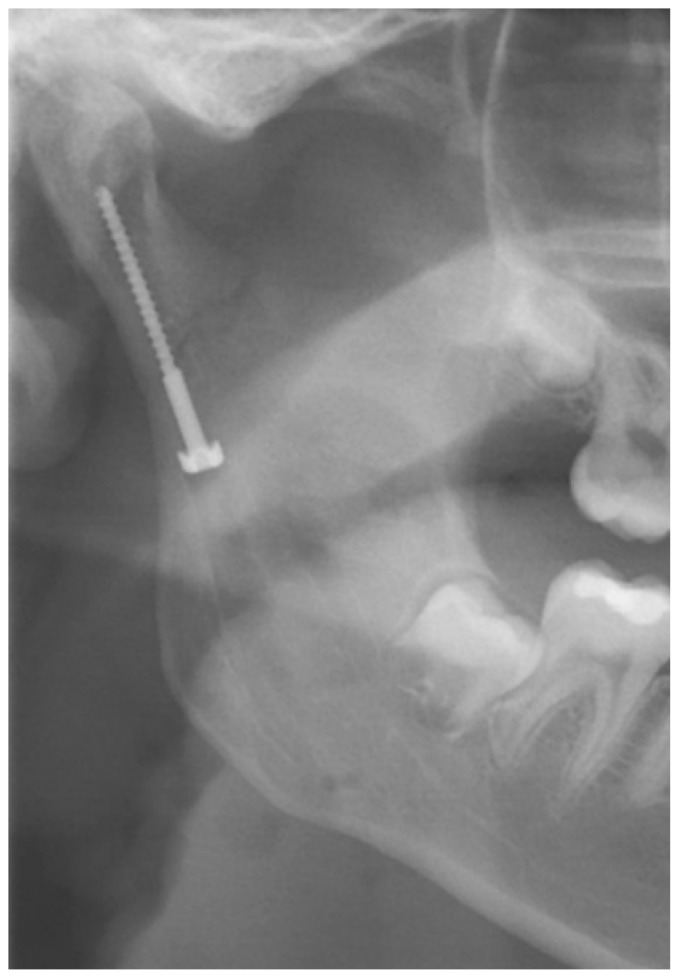
The image shows a Krenkel’s lag screw in one of the patients treated at the authors’ department.

The “classic” submandibular incision, at least 2 cm caudal to the mandible’s lower border, was used for positioning a lag screw, initially described by Eckelt. Krenkel subsequently modified the Eckelt screw, adding a biconcave “washer” for force distribution (Figure 2). After dissection and reduction in the condylar fracture, a channel was drilled on the surface of the posterior border of the ramus ending at the subcondylar area. Starting from this point, the tunnel for the lag screw was drilled in the usual way. Then, the lag screw was placed, engaging the condylar process and thereby fixating the proximal fragment [20,21,22]. Krenkel’s lag screw is no longer available on the market and is of historical interest only.

The “high submandibular” or anteroparotid transmasseteric (APTM) approach is well-described and has the advantage of a minimal prevalence of facial branch injuries [23,24]. Osteosynthesis of the condylar neck/base is performed with a suitable trapezoid condylar plate (TCP) or two straight TriLock Condyle Plates of the System Modus^®^ 2 (Medartis, Basel, Switzerland). Meyer, the inventor of the APTM approach, designed the TCP specifically for this approach [25]. The fractures at the angle, body, and symphysis were exposed through standard transoral incisions or occasionally through a pre-existing skin laceration. Osteosynthesis was performed with straight 2.0 plates of the System Modus^®^ 2 (Medartis, Basel, Switzerland) or lag screws as recommended by the Arbeitsgemeinschaft für Osteosynthese (AO) [26].

### 3.4. Secondary Independent Variables for the Two Cohorts: Surgeons’ Expertise and Procedure Duration

Surprisingly, no significant differences were found regarding the surgeons’ expertise (junior vs. senior) for any of the variables (*p* = 0.684, OR = 0.53, CI: 0.04–3.43).

Operation duration ranged from 105 min to 465 min (mean 192.68 min, SD 84.56 min) in the bottom to top group and from 132 min to 311 min (mean 186.3 min, SD 56.49 min) in the top to bottom group. No significant difference in duration was found between the two cohorts (*p* = 0.682, r = 0.066, CI: 0.00–0.34). Of all patients, 7 (17.07%) had three fractures, and the rest had two fractures. Those with three fractures had an operation duration ranging from 107 min to 465 min (mean 210.57 min, SD 118.2 min), while the operation for those with two fractures lasted from 105 min to 346 min (mean 187.12 min, SD 68.87 min). The odds ratio for patients with two fractures and a suboptimal outcome was 0.49 (CI: 0.07–4.00).

The procedure duration was significantly different between patients with an ideal outcome and those with a suboptimal outcome (*p* = 0.01, r = 0.406, CI: 0.11–0.63), with a mean overall duration of 171.50 min (SD, 57.85 min) and 260.89 min (SD, 102.27 min), respectively. The more complex the case, the longer it takes to treat.

Secondary independent variables are summarized in Table 2 below.

### 3.5. Primary Outcome Measures for the Two Cohorts: Occlusion, Lingual Splay, Neck/Ramus Angle, Redo Surgery, and Need for Occlusal Fine-Tuning

In the bottom-to-top group, 20 (67.52%) patients had an ideal outcome.

Four (12.9%) patients in this group had a substandard surgical outcome.

7 (22.58%) patients had an acceptable outcome.

In the top-to-bottom group, nine patients had an ideal outcome, and one patient had an acceptable outcome.

Three redo operations were necessary in the bottom-to-top cohort.

**Figure 3:**
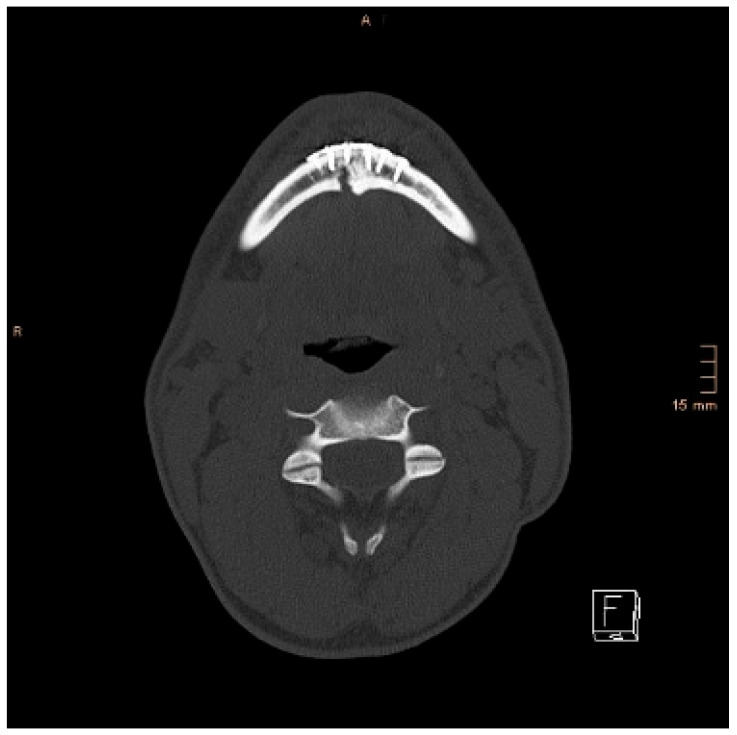
Axial CT scan with splaying of the lingual cortical bone in a symphyseal mandibular fracture after treatment with miniplates.

**Figure 4:**
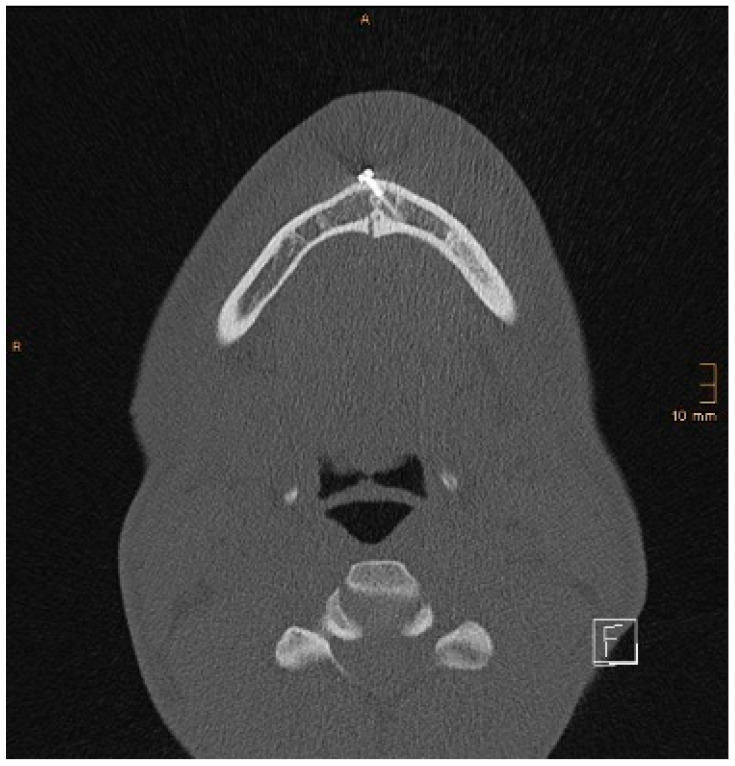
Axial CT scan showing the same patient as in Figure 3 after replacing the miniplates with a lag screw and considerable closure of the lingual gap. However, a small gap in the lingual cortical bone remained, and possibly determined a widening of the mandible.

**Figure 5:**
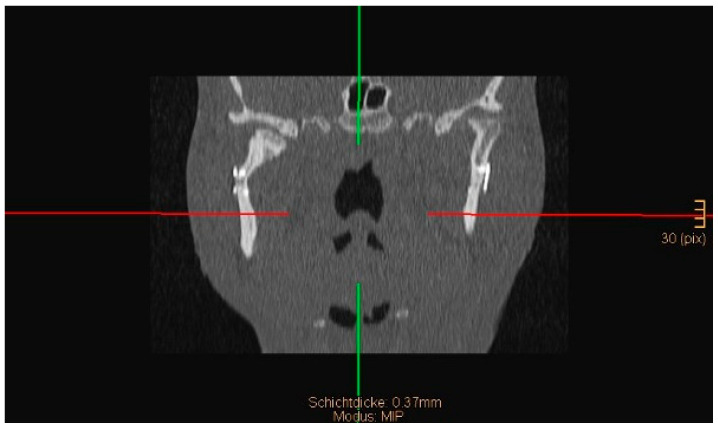
Coronal CT scan showing both condyles after ORIF. A widening of the mandible, caused by a small lingual gap that persisted even after lag screw osteosynthesis at the symphysis, resulted in an angulation of the condylar neck on the right-hand side (same patient as in Figure 3 and Figure 4). The image shown was taken on the third postoperative day after drainage removal and the situation did not worsen over time (last follow-up 1 year postop).

**Figure 6:**
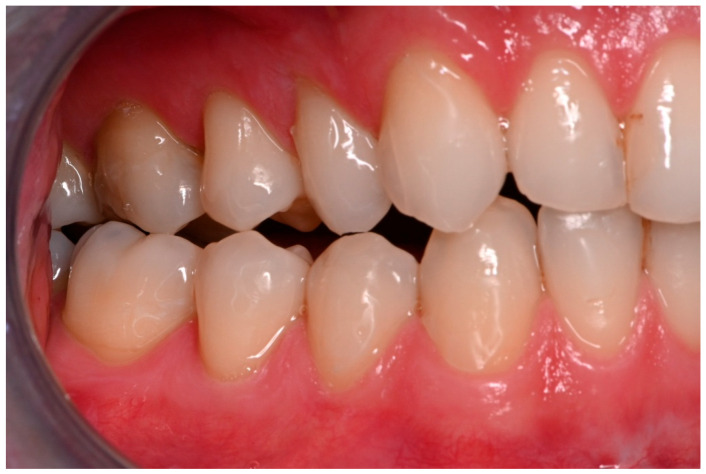
Inadequate occlusion with crossbite on the right-hand side after insufficient reduction in the condylar neck on the same side (see Figure 5).

**Figure 7:**
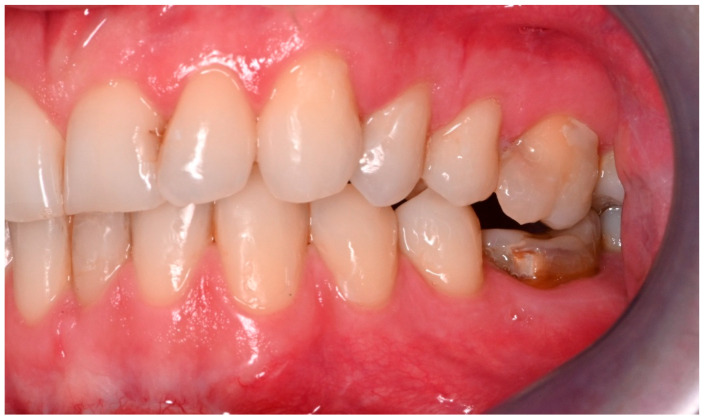
Adequate occlusion on the left-hand side, after satisfactory reduction in the condylar neck on the same side. The lower molar crown was fractured in the accident (see Figure 5).

Two operations had to be redone because of inadequate reposition in the symphysis/parasymphysis with broad (>2 mm) lingual splay. In both cases, straight miniplates were removed. Osteosynthesis was achieved with lag screws. Figure 3 depicts plate osteosynthesis of a symphyseal fracture with a broad lingual gap (Figure 3). Figure 4 shows the same patient after redo operation and osteosynthesis of the same fracture with a lag-screw and a marked reduction in the lingual splaying (Figure 4). Figure 5 demonstrates the condylar reduction in the frontal plane in the same patient. Figure 6 and Figure 7 show the patient’s occlusion (Figure 5, Figure 6 and Figure 7). The third redo operation was due to nonunion and infection of an angular fracture with fracture of the two condylar plates. In this case, after removal of the infected miniplate, the angle fracture was stabilized with a reconstruction plate and a miniplate from an extraoral approach; the two broken miniplates at the condylar process were left in situ.

The fourth patient with a substandard outcome had a neck/ramus angle of 40.5°, required IMF, and selective grinding.

Among the four patients with acceptable outcomes, two had minor occlusal disturbances that resolved in time without any interventions, one patient required IMF, and the last patient had a neck/ramus angle of 39°.

Nine out of ten patients (90%) in the “top to bottom” cohort had an ideal result with anatomical reposition of the fractures and restored occlusion. One patient (10%) had an acceptable result with a lingual splay of 1.7 mm and a need for postoperative IMF. Of note, none required postoperative occlusal interventions.

Observing the association between an optimal/suboptimal outcome and the sequencing yielded no statistically significant result (*p* = 0.231). In other words, there was no significant difference between sequencing and outcome. However, the odds ratio (OR) for a suboptimal outcome regarding the bottom-to-top surgery as opposed to the top-to-bottom surgery was 4.80 (CI: 0.53–236.07). In other words, the odds of having a suboptimal outcome and a bottom-to-top sequence were 4.80 times higher than having a suboptimal outcome and a top-to-bottom sequence. No significant differences were found regarding the lingual splay (<1 mm vs. ≥1 mm) (*p* = 0.433) with an OR of 0.31 (CI: 0.00–26.14). The association between the neck/ramus angle (<10° vs. ≥10°) as well as the occlusion (closed vs. malocclusion) with the sequencing cohorts was not significant either (*p* = 0.564, OR = 0, CI: 0.00–7.74 and *p* = 0.556, OR = 0, CI: 0.00–4.81, respectively). Similarly, the association of the occlusion of closed vs. light open bite vs. premature contact was not significant (*p* = 1.00, Cramer’s V = 0.19, CI: 0.00–0.32). For the overall outcome, no significant difference was found (*p* = 0.119, r = 0.246, CI: 0.04–0.43). The sequencing method also did not show significant associations with occlusal fine-tuning (*p* = 0.165, OR = inf, CI: 0.59-inf) or regarding redo operations (*p* = 0.564, OR = inf, CI: 0.13-inf).

Primary outcome measures are outlined in Table 3 below.

**Figure 8:**
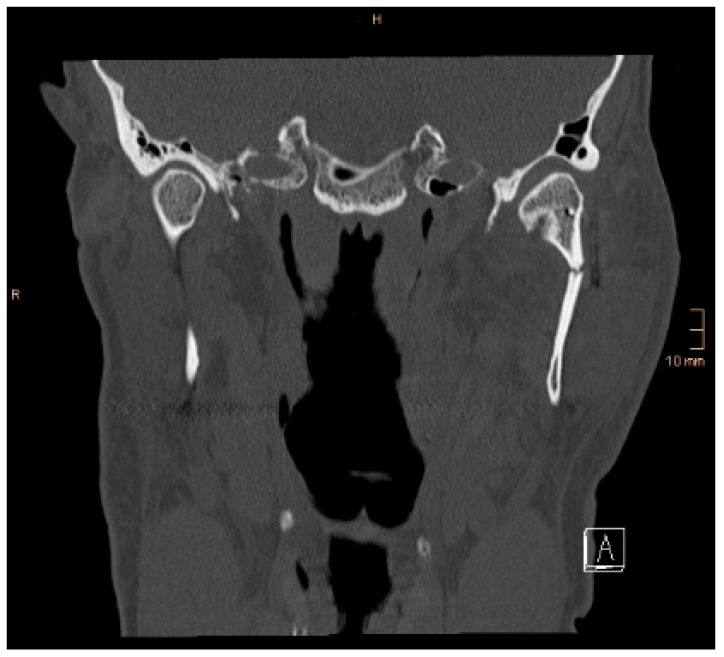
Coronal CT scan showing a condylar neck angulation on the left side after ORIF. An anatomical reduction could not be achieved.

**Figure 9:**
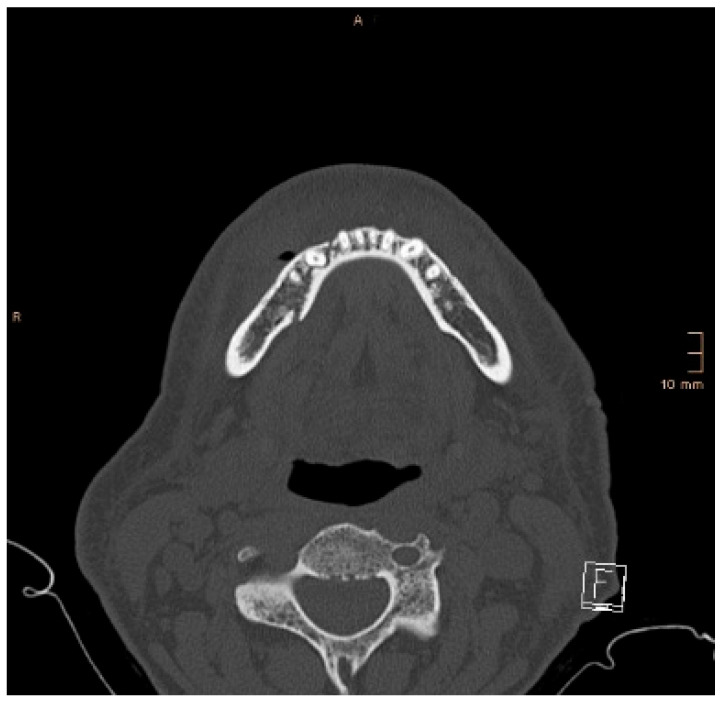
Axial CT scan showing the same patient as in Figure 8. A lingual gap is present after ORIF of the mandibular body fracture, contributing to the inability to reduce the condylar fracture correctly.

Figure 8 and Figure 9 below show another patient with a non-satisfactory reduction in the condylar neck fracture on the left-hand side due to a lingual gap present at a body fracture (Figure 8 and Figure 9).

### 3.6. Secondary Outcome Measures for the Two Cohorts: Non-Occlusal Complications

In the bottom-to-top cohort, the authors observed the following non-occlusal complications, either alone or in combination, in six (19.35%) patients: lower lip/chin hypoesthesia (five patients), wound breakdown (one patient), and weakness of the marginal mandibular branch (two patients). All of the above complications were transient and resolved in time.

In the top-to-bottom cohort, the authors observed the following complications alone or in combination in four (40%) patients: lower lip/chin hypoesthesia (three patients), fistula enoral that resolved after removing the plate (one patient), weakness of the marginal mandibular branch (one patient), and pain referred to the temporomandibular joint (one patient). The above complications resolved in time.

In total, 10 out of the 41 (29.27%) patients had complications. In the bottom-to-top cohort, 6 out of 31 patients (19.35%) had complications. In the top-down cohort, the proportion was 40% (4 out of 10). No association between sequence and occurrence of complications was found (*p* = 0.222). The odds ratio for complications under bottom-to-top surgery as opposed to top-to-bottom surgery is 2.67 (CI: 0.42–16.46).

### 3.7. Multivariable Logistic Regression Analysis

The results of the regression analysis are shown in the table below (Table 4). None of the variables reached statistical significance. Nevertheless, the direction of the estimates is informative: the odds of a suboptimal outcome with a senior surgeon were 0.78 times those with a junior surgeon, and the odds for a suboptimal outcome with the top-down sequencing method were 0.22 times those with the bottom-to-top approach.

## 4. Discussion

The purpose of this study was to identify an “ideal” sequence in ORIF of multifocal mandibular fractures with condylar neck involvement. In doing so, the authors hoped to streamline the operative treatment and improve the outcomes.

The fracture patterns included in the study are isolated (no other non-mandibular additional maxillofacial fractures), multifocal (two or more subsites of which at least one subsite is the condylar basis or neck) mandibular fractures. The authors excluded severely comminuted fractures/defect fractures requiring bone grafting or reconstruction plates, as these are exceptional cases and usually seen in the context of panfacial trauma. The presence of one or two condylar fractures did not alter the approach philosophy, and therefore, the authors did not create subgroups based on bilateral and unilateral condylar fractures. In the authors’ department, condylar head fractures are operated on a case-by-case basis. In the present study, the authors encountered a few cases of multifocal mandibular fractures with condylar head involvement that yielded excellent results and were successfully treated through surgery. However, condylar head fractures were excluded as explained in the exclusion criteria. Nevertheless, the question of the “best” sequencing philosophy remains the same also for condylar head fractures. Regarding the matter of time-to-surgery, all cases were treated within 72 h. As for the surgeons operating on these cases, it was a composite group of staff and trainees with variable levels of expertise, but a senior with adequate knowledge was always either assisting or handling the case. There was no statistically significant difference between juniors and seniors regardless of the statistical tests performed. However, the multivariable logistic regression analysis showed a slight superiority of the more experienced practitioners even if not statistically significant.

Due to the methodology of assessing the lingual gap, the neck-ramus angle, and occlusion in discrete categories, no data are available for computing inter-rater reliabilities. Further studies should investigate this issue in greater detail.

The reason for collapsing different parameters and looking at the “overall surgical outcome” was a practical one: the authors have identified numerous quality indicators and have set a very “high bar” for each of them. Such strict and detailed analysis is possible and useful in clinical studies. However, it is exceptionally rare in clinical practice to achieve a truly “perfect” result. Therefore, despite some suboptimal elements, ultimately, in the clinical arena, a surgeon will have to decide whether the outcome is poor, acceptable, good, or ideal by considering the overall picture.

The literature on treatment sequencing of the above fracture pattern is sparse and controversial. In the “top-to-bottom” field, Nayak et al. reported on four patients with double and triple mandibular fractures involving the condylar process and achieved good outcomes starting the treatment at the condylar level [8]. The same group reviewed a larger series of patients with multifocal mandibular fractures using the same “condyle first” approach [9]. However, the justification for this strategy is unclear. Finally, Zhang et al. proposed starting with the condyle without providing any rationale for their choice [10].

On the other hand, other researchers favored a “bottom-to-top” sequence. Dell’Aversana Orabona et al. conducted two identical retrospective studies dividing mandibular fractures into two groups: fractures of the tooth-bearing areas and fractures of the non-tooth-bearing segments, and identified two groups of patients: group A, which had ORIF of the tooth-bearing fracture first, followed by fixation of the non-tooth-bearing fracture second, and group B, in which the sequence was inverted. The authors concluded that, in their hands, the group with the “bottom-to-top” sequence had fewer “postoperative complications and reduced surgical time and costs”. However, the authors observed that reducing the condylar fracture was easier when addressed first, as the tooth-bearing segment was more mobile [6,7].

In the present analysis, two redo operations out of three were indicated by the presence of lingual splay and subsequent malocclusion, in other words, by inadequate reduction. After multifocal mandibular fractures, there can be an increase in the intergonial distance with facial asymmetry in unilateral fractures or a bilateral facial broadening in bilateral fractures [27,28].

In many cases of multifocal mandibular fractures, the fracture between the angles is produced by direct impact. In contrast, the condylar fracture is indirect, due to buckling of the bone beyond the level of tolerance at a locus minoris resistentiae. The tendency of the angles to displace and stay laterally compared to their original anatomic position in multifocal mandibular fractures is explained by unopposed muscular pull. According to Pedemonte, on the one hand the geniohyoid and the mylohyoid muscle exert a traction on the symphysis/parasymphysis in posterior direction, whereas, on the other, the masseter and the temporal muscles overcome the medial pterygoid muscle and pull the angle and the ramus in a supero-lateral direction, also separating the fracture at the upper border (tension zone) at the body and angle [28].

**Figure 10:**
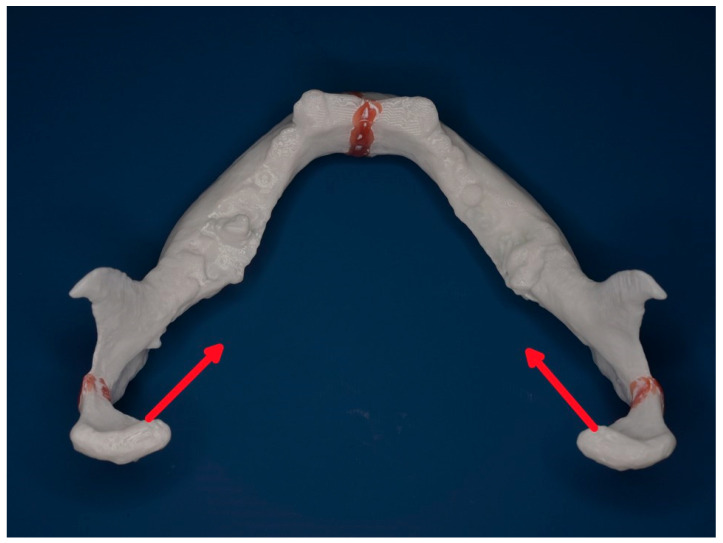
Three-dimensional model with dislocated mandibular fractures (symphyseal, neck right, neck left) on the horizontal plane. Bird’s eye view with insertions of the lateral pterygoid muscles and vectors of dislocation of the condylar process (red arrows).

**Figure 11:**
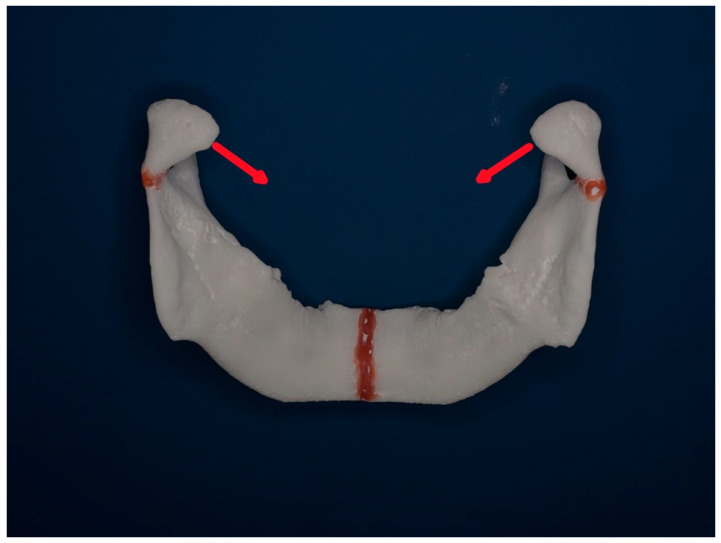
Same model as in Figure 10 seen from the posterior perspective. Red arrows indicate insertions of the lateral pterygoid muscles and vectors of dislocation of the condylar processes in a guardsman fracture.

Figure 10 and Figure 11 below show a mandibular model seen from above and from behind to demonstrate the typical vectors of dislocations of the condylar processes after a neck fracture.

To counter the above, Gerbino et al. start with maxillomandibular fixation, followed by “an intraoral anterior degloving approach allowing visualization of the inferior border and the initial part of the lingual area of the fracture” at the symphysis [11]. Strong manual pressure in medial direction at the angles and simultaneous anterior traction at the symphysis are applied to prevent the “gonial splaying” (increase in the intergonial distance), and two plates stabilize the symphysis [11]. A crucial factor is a “3D” visualization of the symphyseal fracture from at least two different perspectives: anterior and inferior-posterior to avoid plating the fracture with a lingual gap [11]. Alternatively, it is possible to see the inferior and lingual aspect of the mandible with an extraoral submental incision or by using/extending a skin laceration.

As for the type of symphyseal osteosynthesis, two lag screws or two plates, according to Ellis, are equivalent [29]. However, the lag screws are more technique-sensitive.

It is said that the plates can exert a compression on the lingual aspect if they are “overbent” directly in correspondence to the fracture line. In this case, contrary to the general principle of adapting precisely fitting plates on the bone after reduction, the plate is overbent in an arch or a “c shape”, leaving a gap of 2 mm between the anterior aspect of the mandible and the dorsal aspect of the plate. The two ends of the plate rest on the bone. If the plate is sufficiently rigid, the bone will be pulled anteriorly, thereby compressing the fracture at the posterior/lingual aspect and closing the lingual gap [15].

Chen et al., who do not report their sequence, emphasize the importance of removing the hematoma at the line of symphyseal fracture and applying mandibular reduction forceps to achieve anatomical reduction at the lingual aspect [30].

The first author of the present article published a technical note on how to improve outcomes in ORIF of multiple mandibular fractures with condylar involvement [31]. Briefly, four new elements were highlighted: three-dimensional printing, model surgery, Kirschner wires to manipulate the segments in condylar fractures, and a top-to-bottom sequence [31].

In agreement with Schiel et al., the authors find that repositioning the condylar process is less cumbersome if the symphyseal body fracture has not been plated yet, as the hemimandible is easier to manipulate than the entire mandible [32]. Furthermore, once the condylar fracture has been reduced and plated, the hemimandible will be pulled back toward the median by the lateral pterygoid muscle, thereby contributing to actual anatomical reduction without lingual splay [32].

Concerning the hardware, the authors favor the set from Medartis because it is very well thought out, the screws heads are strong and do not easily strip, the thread engages the bone with superb efficacy, and the screws never break. As for the lag screws, the authors used a set that was designed by Krenkel in Salzburg many years ago and is no longer available.

Surprisingly, no significant differences were found regarding the surgeons’ expertise (junior vs. senior) for all variables (*p* = 0.684).

In other words, in our sample, the seniority of the person carrying out the procedure did not impact the outcome.

The procedure duration was significantly different between patients with an ideal and a suboptimal outcome (*p* = 0.01), with a mean overall duration of 171.50 min (SD 57.85 min) and 260.89 min (SD 102.27 min), respectively. The more difficult the case, the longer it takes to treat it.

The present analysis has some limitations: the small sample size, despite starting from a considerable number of mandible fractures, and its retrospective nature.

The reason for enrolling only 10 patients in the top-to-bottom group is that only 10 patients fulfilled the inclusion criteria. The authors acknowledge that this is a small number and that there is a numerical imbalance with the bottom-to-top group, which is considerably larger; The authors strived to create groups with similar fracture patterns that could be compared to each other. There was no other reason for the sequencing other than the surgeon’s preference. The majority of the colleagues followed the “classic” doctrine of the bottom-to-top sequence, also in the authors’ department. Multicenter studies would provide more patients and overcome the limitation of small sample sizes.

The authors performed a power/sample-size consideration to help interpret the wide confidence intervals. Using the observed odds ratio (OR = 4.8) and the standard two-proportion (normal approximation) sample-size formula with α = 0.05 (two-sided) and 80% power yields a required sample size of approximately 43 participants per group to detect an effect of this magnitude. Because the observed sample (10 in the top-to-bottom and 31 in the bottom-to-top group) is smaller than this benchmark, the study is underpowered to estimate the effect precisely, which explains the wide confidence intervals. Therefore, it is advisable to interpret the point estimate cautiously. Future studies aiming to confirm this magnitude of effect should recruit a group of at least approximately 40–50 subjects. Due to the small sample size in the top-to-bottom sequencing group, the statistical power of the hypothesis tests employed is likely limited, despite the use of non-parametric methods. Nevertheless, the findings indicate that the top-to-bottom sequencing yields outcomes that are at least not inferior to those of the bottom-to-top sequencing.

The authors of the present study believe that the “classic” doctrine for addressing non-condylar fractures before condylar fractures originated from an era and a philosophy where all fractures of the condylar process were treated conservatively. This dogma stemmed from the assumption that the condylar region had an almost unlimited remodeling capacity, even without surgery, that surgery was per se detrimental to the vascularity of the condylar process, inducing avascular necrosis, and from the difficulty of achieving anatomical reduction and stable fixation, and the risk of injury to the facial nerve branches. The first author of the present analysis extensively addressed the pros and cons and limitations of the operative and conservative treatments for condylar process fracture in the pediatric and adult population in a multicentric retrospective analysis and a literature review [33,34]. Meanwhile, there is consensus and ample evidence that ORIF for moderately and severely displaced fractures is superior to conservative treatment of condylar process fractures in the adult and pediatric population [33,34].

Anyway, coming back to the specific question that the present analysis tried to address, a prospective randomized controlled multicentric study would provide a higher level of evidence and statistical power.

The strengths of the study include its rigorous analysis and statistical methods, as well as its merit as the first attempt to provide an evidence-based answer to a relevant clinical question that has not been adequately addressed to date.

### Take-Home Points for the Practice

1. Plan carefully before the operation. Nowadays, virtual 3D reconstructions are readily available in imaging software and provide a handy aid for visualizing fractures. This tool, in turn, helps in selecting the best possible approach for the condylar process.
2. Physical 3D models in 1:1 scale can be valuable for planning as the surgeon can observe the fragments in detail, developing a better understanding of the orientation of the fragments, which is most useful during the procedure. Furthermore, hardware can be selected and pre-bent [31].
3. The surgical team should consist of three people, and at least one should be a senior.
4. If possible, treat all the fractures that need ORIF in one single operation, exposing all the fractures before reducing and plating them. These injuries should ideally be treated as semi-elective cases at the start of the day when the teams in the operating theater are fresh, time is not a concern, and more hands are available in case of difficulties. On the contrary, addressing only the dentate “more urgent” and easier fracture first, approaching the condylar fracture at a second stage, frequently implies the necessity of redoing the osteosynthesis at the symphysis/parasymphysis because of the lingual splaying that hinders an anatomic reposition of the condylar process. Suppose that active bleeding can be stopped and a mobile dentate fracture is sufficiently stabilized with an interdental ligature. In that case, clinical practice has shown that with antibiotic coverage, there is no need for immediate ORIF in standard mandibular fractures communicating with the oral cavity [35].
5. If possible, prefer a top-to-bottom sequencing during ORIF. In the top-to-bottom sequence, the non-condylar fractures are not osteosynthesized before addressing the condylar fracture. Therefore, the surgeon has the mechanical advantage of manipulating a smaller distal segment instead of the whole mandible. Critically, the patients should be fully paralyzed to minimize muscle pull. On the other hand, the surgeon cannot rely on dental occlusion but must plate the condylar fracture only based on the best possible fitting of the fracture lines. Furthermore, the manipulation of the distal segment should be careful not to damage the inferior alveolar nerve in case of dislocation of a body or angular fracture.
6. Take a short break in lengthy and complicated operations if an adequate reduction appears impossible despite all efforts. Leave the operating room, hydrate yourself, and reassess the situation, considering both the imaging and the clinical findings. Taking a step back from the problem at hand and walking a few steps can both decrease stress and enhance cognitive ability [36]. Robert Acland, a very accomplished microvascular surgeon, used to recommend taking a break in long operations to avoid fatigue, stubborn insistence, and desperation in case of difficulties [37].
7. Ask the anesthetist to administer neuromuscular blocking agents to achieve complete muscle relaxation once the dissection is deeper than the plane of the facial nerve branches or after their identification. Complete muscular relaxation facilitates adequate reduction and drastically reduces the physical efforts for the surgical team.
8. Delayed cases can be more difficult: make sure to entirely remove the hematoma/callus in the line of fracture.
9. The surgeon must assess the quality of the reposition from at least two different perspectives during the surgery.
10. If available, utilize an intraoperative CT scan for quality control. Intraoperative imaging may avoid a second general anesthesia if the reduction is not sufficient.
11. It is very easy and most useful to palpate the lingual mandibular contour through the floor of the mouth and feel if a step is present. Haptic or tactile feedback provides vital information when reducing and plating the mandible at the symphysis, parasymphysis, and body, even if the lingual aspect is not directly visualized.
12. To eliminate the lingual step and improve the precision of the reduction, one assistant can exert medial pressure on the mandible body at the level of the angles, and another assistant applies a traction in the anterior direction on the lingual aspect at the symphysis using a zygomatic bone hook. Reduction forceps can be used for temporary stabilization during osteosynthesis of the symphysis and parasymphysis.
13. A transcutaneous approach to the symphyseal area—sometimes possible through a skin laceration on the chin—allows excellent visibility and increases the chance of ideal osteosynthesis without a lingual gap. Alternatively, a small submental skin incision “hidden” behind the chin in this safe area can provide a more advantageous perspective.
14. Lag screws are technique-sensitive and more difficult than plates. However, they can be most helpful for achieving anatomical reduction or at least the best possible reduction at the symphysis/parasymphysis, especially in cases where mandibular widening is expected because of the fracture pattern.
15. Debrief: reflection on less-than-ideal results—better as a team—is a painful process that can bring fruit for the future.

## 5. Conclusions

Suppose the reduction in each fracture is anatomical. In that case, ORIF sequencing does not influence the outcome, as when putting a jigsaw puzzle together, so there is no reason for a dogmatic approach. However, within the limitations of the study, based on our results, comparing the two cohorts, a top-to-bottom ORIF sequence is a favorable factor for achieving better outcomes. This positive influence stems from the reduced possibility of plating the non-condylar fracture with a lingual gap in the top-to-bottom sequence compared to the bottom-to-top sequence. The above effect derives from the fact that after reduction and stabilization of the condylar process, the lateral pterygoid muscle can exert its traction in an antero/medial direction on the whole hemimandible, thereby pulling it towards the median and closing the lingual gap at the symphysis/parasymphysis. In other words, the tension exerted by the lateral pterygoid muscle helps in closing the lingual gap at the symphysis and represents the biomechanical rationale for fixating condylar fractures first. Therefore, the traction of the lateral pterygoid muscle is the surgeon’s enemy because it dislocates the condylar process before ORIF. After ORIF, it becomes his/her ally because it closes the lingual gap in multiple mandibular fractures with condylar involvement.

## Figures and Tables

**Table 1:** Patients’ demographics.

Demographics		Bottom-to-Top (31)	Top-to-Bottom (10)	*p*-Value
Age in years mean (SD)		40.81 (16.53)	37.30 (18.46)	0.543
Sex				1
	f	7 (22.58%)	2 (20%)	
	m	24 (77.42%)	8 (80%)	
Etiology				0.205
	Assault	5 (16.13%)	3 (30%)	
	Fall	8 (25.81%)	1 (10%)	
	RTA	18 (58.06%)	5 (50%)	
	Work accident	0 (0%)	1 (10%)	
Number of fractures mean (SD)		2.2 (0.4)	2.1 (0.32)	0.514

**Table 2:** Secondary independent variables: surgeons’ expertise and procedures’ duration.

		Bottom-to-Top (31)	Top-to-Bottom (10)	*p*-Value
Duration mean (SD) (min)		192.68 (84.56)	186.30 (56.49)	0.68
expertise				0.68
	junior	10 (32.26%)	2 (20%)	
	senior	21 (67.74%)	8 (80%)	

**Table 3:** Primary outcome measures: ramus/neck angle, lingual gap, occlusion, and redo surgery.

		Bottom-to-Top (31)	Top-to-Bottom (10)	*p*-Value
occlusion				1
	closed	27 (87.1%)	10 (100%)	
	light open bite	2 (6.45%)		
	premature contact	2 (6.45%)		
Lingual splay				0.433
	<1 mm	30 (96.77%)	9 (90%)	
	≥1 mm	1 (3.23%)	1 (10%)	
Neck/ramus angle				0.330
	10–40°	1 (3.23%)		
	<10°	28 (90.32%)	10 (100%)	
	>40°	2 (6.45%)		
Overall outcome				0.119
	acceptable	7 (22.58%)	1 (10%)	
	ideal	20 (67.52%)	9 (90%)	
	substandard	4 (12.90%)		
Occlusal fine-tuning				0.165
	no	23 (74.19%)	10 (100%)	
	yes	8 (25.81%)		
Redo operation				0.564
	no	28 (90.32%)	10 (100%)	
	yes	3 (9.68%)		

**Table 4:** Multivariate logistics regression model concerning expertise, fractures number and sequencing.

Variable	OR	95% CI	*p*-Value
Expertise (senior)	0.78	0.17–3.60	0.751
Fractures number	1.95	0.34–11.33	0.68
Sequencing method (top-to-bottom)	0.22	0.02–2.00	0.179

## Data Availability

Additional data are available upon request to the corresponding author.
